# Supplementary material for: COVID-19: viral–host interactome analyzed by network based-approach model to study pathogenesis of SARS-CoV-2 infection
Source: J Transl Med. 2020 Jun 10;18:233. doi: 10.1186/s12967-020-02405-w (PMC7286221; doi:10.1186/s12967-020-02405-w)
Supplement: Supplementary file 2 — Additional file 2: Figure S1. Pairwise distances along 259 full length CoV genomes. In the bottom of picture, indicative gene positioning along CoVs genomes is reported. The list of all considered genomes is reported in Additional file 1: Table S1. Figure S2. 3D structure of S-glycoprotein of SARS-CoV-2 and comparison with the ortholog from HCoV-229E, SARS-CoV, and MERS-CoV. Lateral (a) and superior (b) representation of SARS-CoV-2 S-glycoprotein, deducted for the sequence of patient INMI1 (MT066156.1). Each subunit chain has a different color. Structure comparison of S-glycoprotein subunit between: HCoV-229E and SARS-CoV-2, in purple and blue respectively (c); SARS-CoV and SARS-CoV-2, in red and blue, respectively (d); MERS-CoV and SARS-CoV-2, in green and blue, respectively (e). Figure S3. Amino acid alignment and secondary motifs in the receptor binding domain (RBD) of S-glycoprotein of HCoV-229E, SARS-CoV, MERS-CoV and SARS-CoV-2 are shown. Legend of secondary motifs identifiers: H = α Helix, E = β Sheet, X = Random coil. Figure S4. HCoV-229E–host interactome resulting from RWR applied to the top 200 closest proteins identified by RWR, using S-glycoprotein of HCoV-229E. Figure S5. SARS-CoV–host interactome resulting from RWR applied to the top 200 closest proteins identified by RWR, using S-glycoprotein of SARS-CoV. Figure S6. MERS-CoV–host interactome resulting from RWR applied to the top 200 closest proteins identified by RWR, using S-glycoprotein of MERS-CoV. [file 12967_2020_2405_MOESM2_ESM.docx]

**Appendix**

To evaluate the diversity along full CoV genome, 259 sequences of CoVs, infecting different hosts were retrieved from GenBank and aligned. The diversity was evaluated on full-length genomes as pairwise distance, shown in Figure S1**.**

For 3D S-glycoprotein representation, Among the models obtained with the Swiss PDB viewer, we selected the version built with the sequence (RCSB-PDB 6VSB) showing the maximum similarity (99.58%) with the query sequence (INMI-1) and the best QMEN value (-2.65) (Figure S2).

For the alignment RBD in four CoVs, similarity in the secondary structure was shown in overall sequences, especially in SARS-CoV-2 and SARS-CoV (Figure S3).

To simulate biological responses and compare it with clinical outcomes during the infections of both viral species, it was chosen only S-glycoprotein, requesting 200 the closest protein to seed protein in interactome.

For HCoV-229E, a homogeneous PPI network cloud was reconstructed, highlighting the direct interaction between S-glycoprotein and ANPEP (score 0.21), followed by RAD18 (score 0.016327) and APEX1 (score0.016304), associated to DNA repair and regulation of transcriptional factors (Figure S4).

For SARS-CoV, early interactions in the PPI network were between S-glycoprotein and CD209 (score 0.041782), followed by CLEC4M (score 0.039022) and SFTPD (score 0.036978), CLEC4G (score 0.036391), EIF3F (score 0.036083), ACE2 (score 0.036038), which are considered virus-recognition receptors, except for EIF3F (Figure S5).

When S-glycoprotein of MERS-CoV has been used as seed for RWR, PPI network reviled the highest proximity with DPP4 (score 0.2140), receptor for MERS-CoV, but followed by CXCL11 (score 0.00391), CCL11 (score 0.00389) and CXCL9 (score0.00389), CXCL10 (score 0.00382), CXCL2 (score 0.00389), highlighted immediate and massive activation of chemokine pathways (Figure S6).


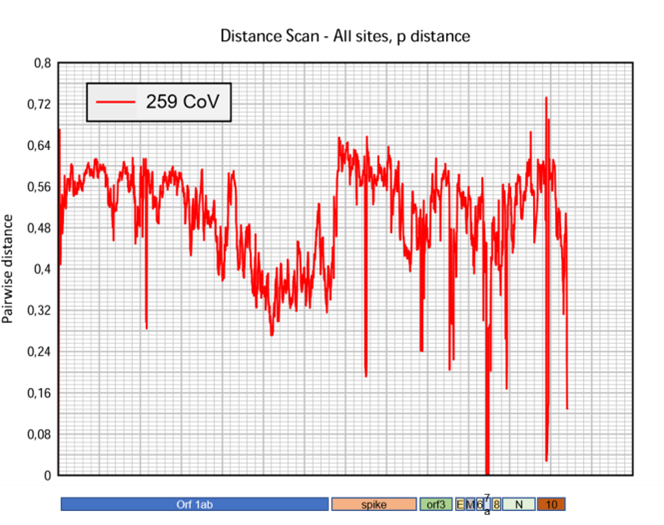


Figure S1. Pairwise distances along 259 full length CoV genomes. In the bottom of picture, indicative gene positioning along CoVs genomes is reported. The list of all considered genomes is reported in Table S1.


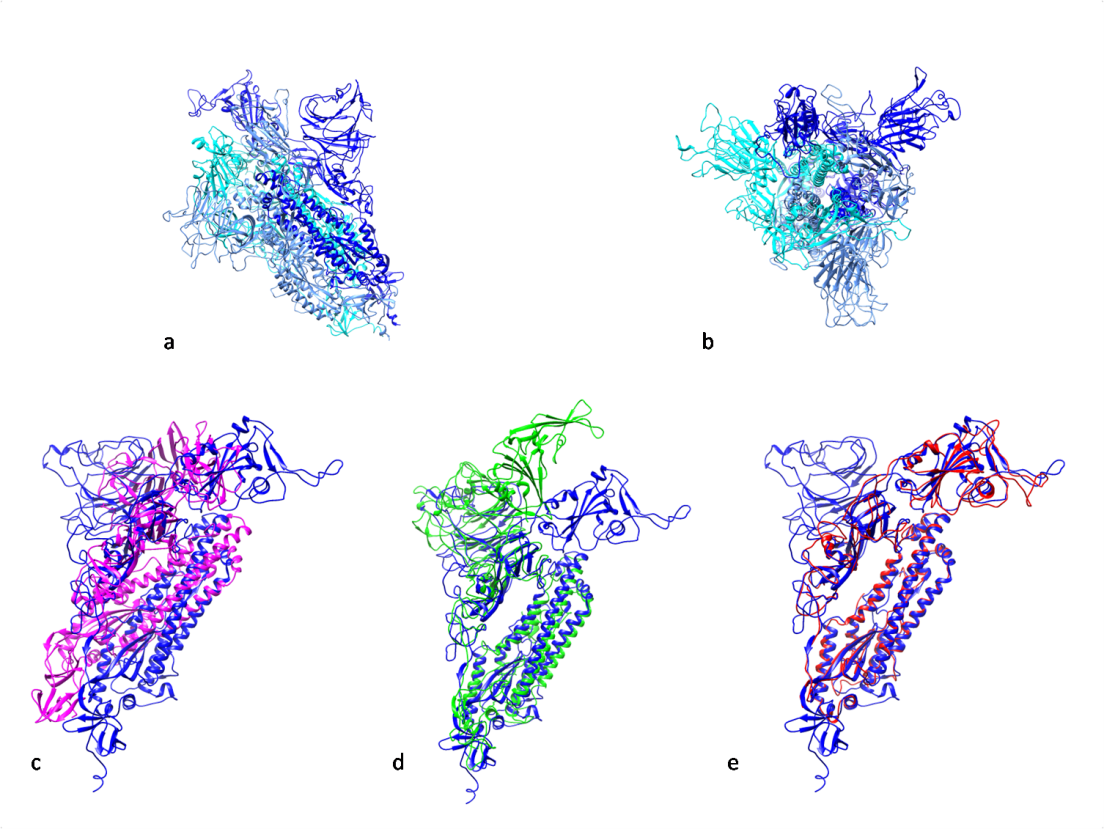


Figure S2. 3D structure of S-glycoprotein of SARS-CoV-2 and comparison with the ortholog from HCoV-229E, SARS-CoV, and MERS-CoV. Lateral (a) and superior (b) representation of SARS-CoV-2 S-glycoprotein, deducted for the sequence of patient INMI1 (MT066156.1). Each subunit chain has a different color. Structure comparison of S-glycoprotein subunit between: HCoV-229E and SARS-CoV-2, in purple and blue respectively (c); SARS-CoV and SARS-CoV-2, in red and blue, respectively (d); MERS-CoV and SARS-CoV-2, in green and blue, respectively (e).

Figure S3**.**Amino acid alignment and secondary motifs in the receptor binding domain (RBD) of S-glycoprotein of HCoV-229E, SARS-CoV, MERS-CoV and SARS-CoV-2 are shown. Legend of secondary motifs identifiers: H = α Helix, E = β Sheet, X = Random coil.


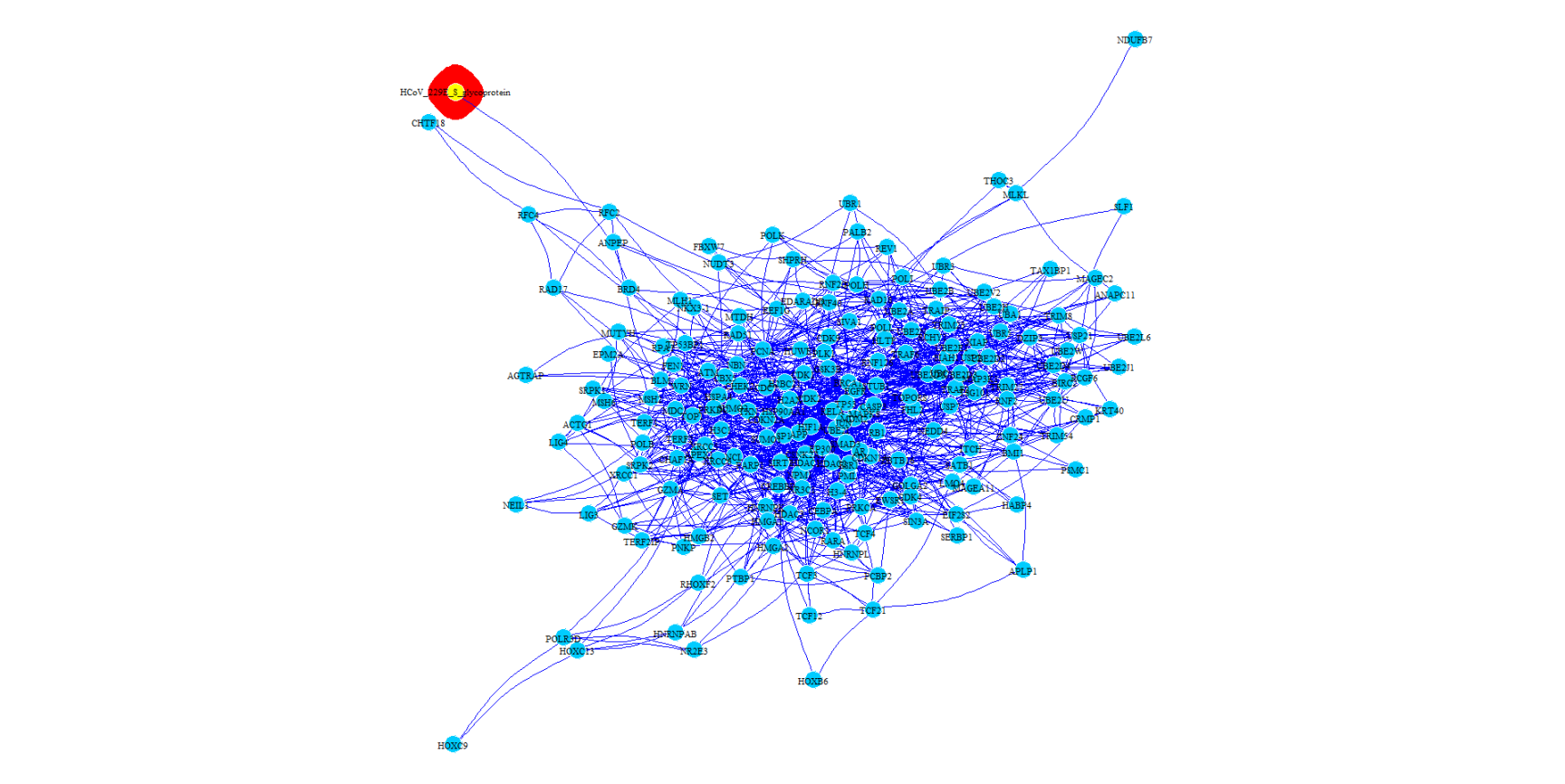


Figure S4**.** HCoV-229E - host interactome resulting from RWR applied to the top 200 closest proteins identified by RWR, using S-glycoprotein of HCoV-229E.


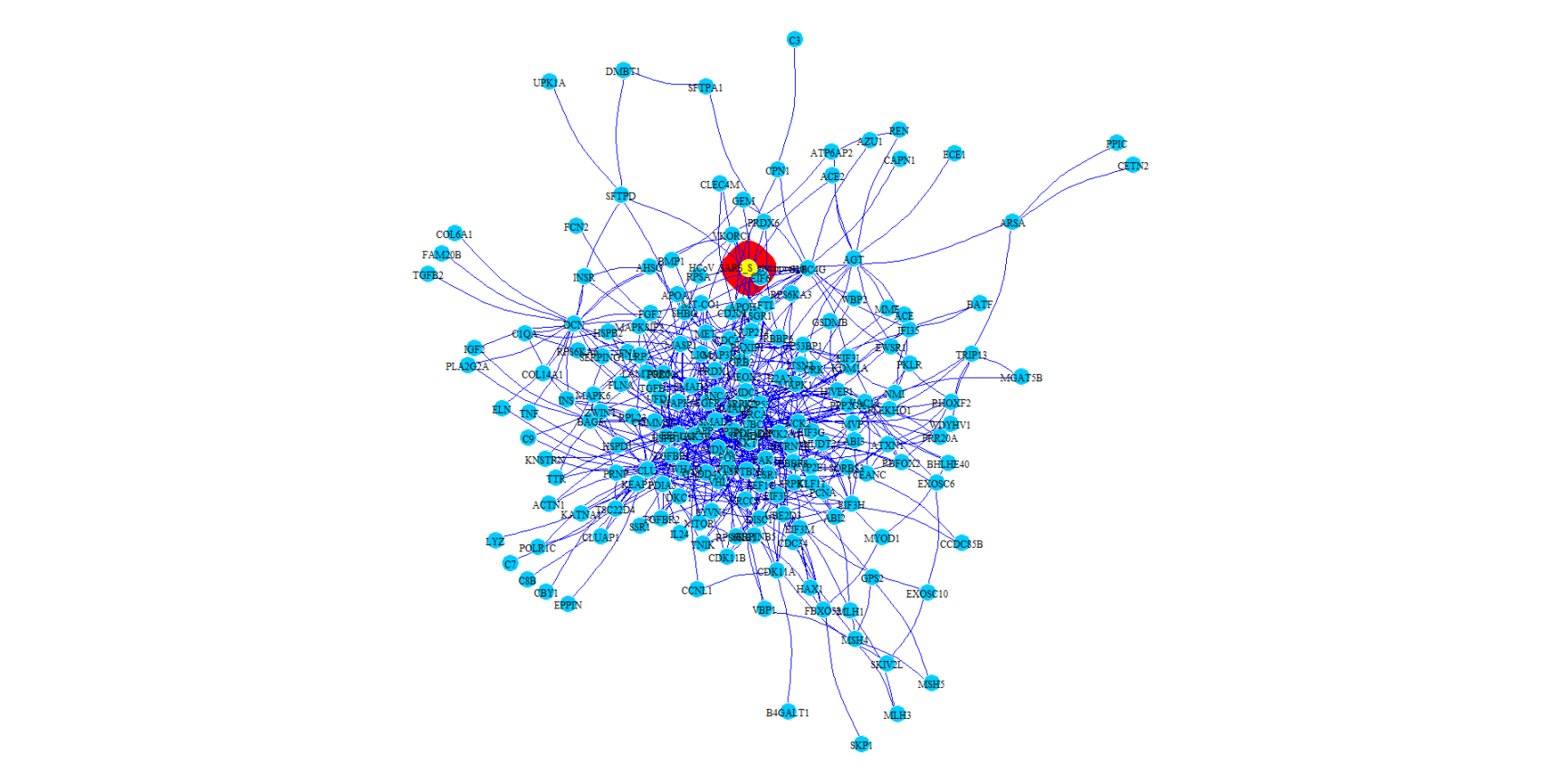


Figure S5. SARS-CoV - host interactome resulting from RWR applied to the top 200 closest proteins identified by RWR, using S-glycoprotein of SARS-CoV.


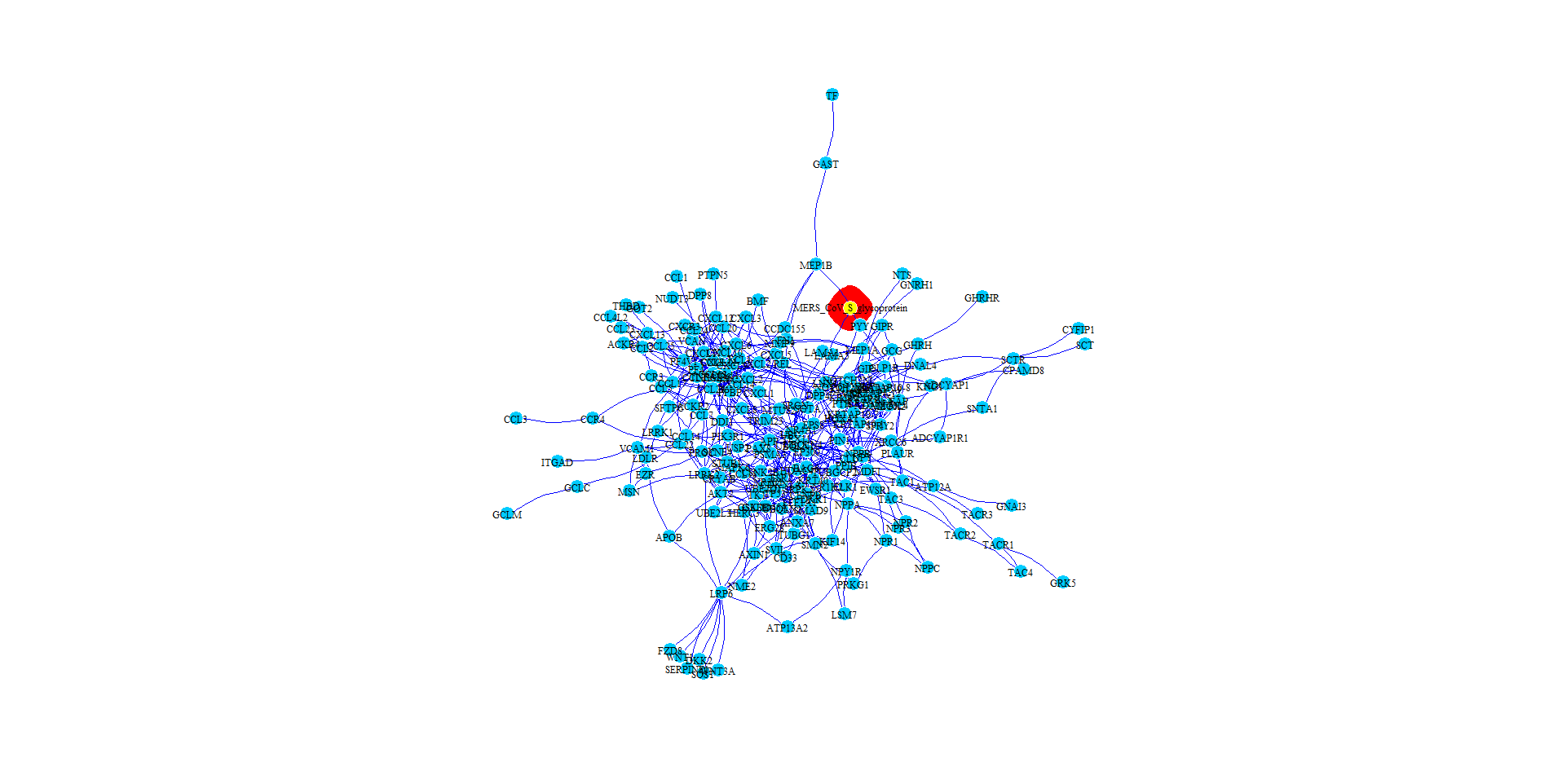


Figure S6. MERS-CoV - host interactome resulting from RWR applied to the top 200 closest proteins identified by RWR, using S-glycoprotein of MERS-CoV.
